# Ptomaines

**Published:** 1888-10

**Authors:** Charles Mayr

**Affiliations:** Springfild, Mass.


### PTOMAINES.

BY PROF. CHAS. MAYR, SPRINGFILD, MASS.

READ BEFORE THE UNION MEETING AT BOSTON, JULY 11, 1888. ABSTRACT BY IRA G. BAUMGARDNER, D.D.S., PHILADELPHIA.

Prof. Mayr began his address by referring to the danger run by physicians of inoculating themselves in making autopsies, and performing surgical operations in certain cases. He said that the theory which had held sway for many years of a chemical poison had been disproven. It is not the action of a chemical poison in these cases, but of an infectious agent which enters through the wound, and afterwards develops within the body. These micro-organisms in their growth throw off certain waste products which are called ptomaines. A very large number have been tabulated. Other products of life force have been discovered, as, for instance, leucomaines—a substance produced during digestion, and other physiological actions throughout the body. But leucomaines have not been sufficiently isolated and identified to speak of them as distinct chemical individuals; for we can speak of chemical individuals just as well as individuals in mineralogy. Leucomaines have not been, as yet, scientifically individualized, therefore are supposititious bodies. Ptomaines on the other hand are distinct chemical entities. If any of you have had any experience in photography you know that, in making "blue prints," the color is produced by the reducing action of the solar rays. Some ptomaines have the same reducing power. If a few drops of the chemical mixture used in photography in making blue prints are allowed to fall into a mixture containing ptomaines the solution will immediately turn blue.

It has been claimed that all retrograde processes in the human body are putrefactive; even the production of alcohol can be proven to be a putrefactive change. It is merely "a hair splitting of words" to call one fermentation and the other putrefaction. Prof. Brieger took five hundred pounds of horse-flesh, and allowed it to putrefy for two weeks, after which he obtained $\frac{1}{4}$ of an ounce of ptomaines. The most abundant ptomaine found by Brieger was termed *neuridine*, which when isolated resembles somewhat saltpetre crystals. It is entirely harmless. The next compound discovered as a result of the putrefactive process he termed *cadaverine*, another *putricine* and so on, each name denoting its source. These are all comparatively harmless. The only one isolated that was poisonous was termed *neurine*. He estimated that it would take $\frac{1}{4}$ of a grain of this to kill a human being. Brieger has also investigated the waste products of several disease gems; notably that of typhoid fever in which he discovered a specific poison, which he denominated *typhotoxine*, suggesting that it was a toxical product. Prof. Mayr considers this Brieger's most important discovery. He allowed the germ of typhoid fever to act upon raw meat and obtained the poisonous principle from the extract. He was enabled by experimentation upon rabbits and guinea pigs to produce many of the typhoid symptoms. Other symptoms present he attributed to vital reaction. Brieger carried his experiments into the realm of tetanus. It has been found that lockjaw is produced by a specific micro-organism which enters the system by means of a wound, sometimes that of a rusty nail, which makes an ugly wound that does not heal readily. Brieger obtained from the product of this germ a new ptomaine called by him *tetanotoxine*, which is produced in the circulation by the germs.

He investigated certain clams, called mytilus, found on the English and Continental shores, and very similar to our Long Neck clams. One day fifty persons became sick from eating clams procured from the North sea. Some of these were sent Dr. Breiger, and he discovered a poison which he called *mytilotoxine*, which could only be procured in very small quantities, but which was very intoxicating, fully 120 times as much so as alcohol. The first effect, when poisoned by it, is an excessive exhiliration; soon, however, followed by great depression, in which stage many die.

In making these investigations the substance is allowed to putrefy—then the fluid is filtered out and concentrated and precipitated by bichloride of mercury. All the ptomaines combine more or less readily with sublimate and are liberated by sulphurated hydrogen. The most efficient antidote yet discovered for neurine and tetanotoxine is atropia. The symptoms of neurine poisoning in rabbits appeared very rapidly after the administration of the drug. The secretions of the salivary glands were markedly increased; great tears would drop down from their eyes, and in a short time they died without seeming to suffer any pain. These chemical compounds are seemingly very unstable. In making experiments upon putrescent masses, it was found that the ptomaines were most abundant at the end of one week; much less so at the close of two weeks, and that only ammonia remained after three weeks of putrefaction. In answer to a question from Dr. Atkinson as to whether any nitrogen entered into the composition of ptomaines, Prof. Mayr said: They are in their constitution ammonium bases—ammonia has the composition $NH_3$, that is one cubic foot of nitrogen is combined with three cubic feet of hydrogen gas. If united with one cubic foot of hydrogen, we would have an organic ammonia, called mono-ethylanime, and thus by substituting one hydrogen after another by an organic radical we obtain all these organic alkaloids. The compositions of these complicated ptomaines has been very well established; for instance,

*neuridine* is represented by $C_5H_4N_2$. It is interesting to know that most chemical compounds found in the body are ethyl compounds; they contain the common radicals of alcohol. In conclusion the essayist said: It is true that these facts are not directly transferable into dollars and cents in daily practice. I did not present them for that purpose, but with the view of interesting you in problems that belong to a wider sphere. It is only by reaching outside of our own walls that we enlarge our boundaries.

